# Effect of Acipimox on Plasma Lipids and Glucose/Insulin in Pregnant Rats

**DOI:** 10.1080/15604280214938

**Published:** 2002

**Authors:** I. Sánchez-Vera, B. Bonet, M. Viana, E. Herrera, A. Indart

**Affiliations:** 1 Facultad de Ciencias Experimentales y de la Salud Universidad San Pablo–CEU Madrid Spain; 2 Departamento de Pediatría y Neonatología Fundación Hospital Alcorcón C/Budapest 1, Alcorcón Madrid 28922 Spain

## Abstract

To determine how a reduction in maternal hypertriglyceridemia during late pregnancy may affect glucose/insulin relationships, pregnant and virgin rats were orally treated with acipimox, a potent antilipolytic agent. In 20-day pregnant rats receiving 80 mg of acipimox, plasma triglycerides (TG), free fatty acids (FFA), and glycerol decreased more than in virgin rats shortly after the drug (up to 7 hours), when compared with animals treated with distilled water, whereas plasma glucose level was unaffected by the treatment in either group of rats. When acipimox was given every 12 hours from day 17 to day 20 of pregnancy, plasma TG, FFA, and glycerol levels progressively increased, whereas they either decreased or did not change in virgin rats receiving the same treatment, with no effect in plasma glucose levels in either group. Fetal body weight was lower than in controls in 20-day pregnant rats that received acipimox for 3 days. On day 20 of pregnancy, 3 hours after receiving acipimox or distilled water, rats received a 2 g glucose/kg oral load and it was found that the change in plasma glucose was similar in both groups, whereas the increase in plasma insulin was greater in pregnant rats treated with acipimox. However, no difference was found in either variable after the oral glucose load in virgin rats receiving acipimox or distilled water. No differences in plasma glucose levels were found after intravenous (IV) administration of insulin in pregnant rats treated or not treated with acipimox. In conclusion, present results show that administration of acipimox during the last days of gestation inhibited lipolysis and decreased fetal weight. Over a short period of time, in pregnant rats, reductions of plasma FFA and TG after acipimox treatment improved the glucose-induced insulin release, but did not seem to have any effect in peripheral insulin resistance.


*Int. Jnl. Experimental Diab. Res.*, 3:233–239, 2002
Copyright © 2002 Taylor & Francis
1560-4284/02 $12.00 + .00
DOI: 10.1080/15604280290013973

Taylor & Francis healthsciences

### Effect of Acipimox on Plasma Lipids and Glucose/Insulin in Pregnant Rats

**I. Sánchez-Vera,[1] B. Bonet,[1,2] M. Viana,[1] E. Herrera,[1] and A. Indart[1]**

[1] *Facultad de Ciencias Experimentales y de la Salud, Universidad San Pablo–CEU, Madrid, Spain*
[2] *Departamento de Pediatría y Neonatología, Fundación Hospital Alcorcón, Madrid, Spain*

**To determine how a reduction in maternal hypertriglyceridemia during late pregnancy may affect glucose/insulin relationships, pregnant and virgin rats were orally treated with acipimox, a potent antilipolytic agent. In 20-day pregnant rats receiving 80 mg of acipimox, plasma triglycerides (TG), free fatty acids (FFA), and glycerol decreased more than in virgin rats shortly after the drug (up to 7 hours), when compared with animals treated with distilled water, whereas plasma glucose level was unaffected by the treatment in either group of rats. When acipimox was given every 12 hours from day 17 to day 20 of pregnancy, plasma TG, FFA, and glycerol levels progressively increased, whereas they either decreased or did not change in virgin rats receiving the same treatment, with no effect in plasma glucose levels in either group. Fetal body weight was lower than in controls in 20-day pregnant rats that received acipimox for 3 days. On day 20 of pregnancy, 3 hours after receiving acipimox or distilled water, rats received a 2 g glucose/kg oral load and it was found that the change in plasma glucose was similar in both groups, whereas the increase in plasma insulin was greater in pregnant rats treated with acipimox. However, no difference was found in either variable after the oral glucose load in virgin rats receiving acipimox or distilled water. No differences in plasma glucose levels were found after intravenous (IV) administration of insulin in pregnant rats treated or not treated with acipimox. In conclusion, present results show that administration of acipimox during the last days of gestation inhibited lipolysis and decreased fetal weight. Over a short period of time, in pregnant rats, reductions of plasma FFA and TG after acipimox treatment improved the glucose-induced insulin release, but did not seem to have any effect in peripheral insulin resistance.**

Received April 2002; accepted August 2002.

The authors thank Pharmacia & Upjohn for supplying the acipimox. This work was supported by a Grant from Universidad San Pablo–CEU (6/96).

Address correspondence to Dr. B. Bonet, Servicio de Pediatría, Fundación Hospital Alcorcón, C/Budapest 1, 28922 Alcorcón, Madrid, Spain. E-mail: bbjbonet@fhalcorcon.es

**Keywords** Acipimox; Fetal Weight; Lipolysis; Pregnancy; Triglycerides

The enhanced lipolytic activity present during late gestation gives rise to an elevation in plasma free fatty acids (FFA) and triglycerides (TG), both in women and rats [1–3]. Some tissues use fatty acids as fuel, sparing glucose for the fast-growing fetus and those maternal tissues that can only use glucose as fuel energy [4]. The mechanisms involved in this elevated lipolytic activity are uncertain, but the rising levels of placental lactogen and the insulin resistance during late pregnancy seem to play a relevant role [5–7].

The enhanced availability of FFA to the liver seems to contribute to the increased production of TG by this organ [8] and therefore to the development of maternal hypertriglyceridemia. Exaggerated increment of TG in maternal circulation may be involved in some of the pregnancy-induced complications, including eclampsia [9] and insulin resistance that would cause the development of gestational diabetes [7], 2 of the most common pregnancy-induced complications. Therefore, decreasing the hypertriglyceridemia of late pregnancy may be of therapeutic use. Nevertheless, this therapeutic approach may not be risk free for the fetus, because in human pregnancies maternal TG have a positive correlation with the newborn weight [10], and decreased lipid levels have been associated with intrauterine growth retardation [11]. To test how a decline in FFA and TG during late pregnancy affects maternal glucose/insulin relations, we studied the effects of acipimox, a powerful inhibitor of lipolysis [12–14] in late pregnant rats, when adipose tissue lipolytic activity is enhanced.

### METHODS AND EXPERIMENTAL DESIGN

### Animals

Female virgin Wistar rats from our own colony, weighing 190 to 220 g, were housed in a temperature-controlled room (22°C + 1°C) with alternating 12-hour light and dark cycles, and fed a Purina Chow diet (Rat and Mouse standard diet, Beekay Feeds, B.K. Universal S.L., Barcelona, Spain). The care and handling of the animals throughout the study followed the current law for Animal Care of the European Community (Strasburg, March 18, 1986). The rats were mated and the day that sperm appeared in the vaginal smears was considered day 0 of gestation, when rats were divided into 2 different experimental groups.

### Experiment 1

To determine the hypolipemic effects of acipimox during late pregnancy, 20-day pregnant rats were divided in 2 experimental groups. Rats from 1 group received by gavage 80 mg of acipimox (Farmitalia Carlo Erba, Milano, Italy) diluted in 0.3 mL of distilled water, whereas those from the second group received the same amount of distilled water. Two groups of virgin rats were also studied in parallel under the same experimental conditions. From the moment of treatment, the animals were kept fasted and blood samples were obtained before and 1.5, 3, 5, and 7 hours thereafter. FFA, glycerol, TG, and glucose were determined.

### Experiment 2

To study whether the daily administration of acipimox from days 17 to 20 of pregnancy, a period of high lipolytic activity, was able to decrease plasma levels of TG and modify fetal growth, 2 groups of rats were used. One group received 80 mg of acipimox as above at 9:00 and 21:00 hours during the 3 days of the study. The second group received distilled water as a control. Two groups of nonpregnant animals were also studied, in parallel, under the same experimental conditions. No difference in weight gain between 17 and 20 days of pregnancy was observed in rats treated with acipimox compared with distilled water. Tail blood was obtained daily between 8:00 and 9:00 AM, just before the morning treatment, whereas on day 20, the animals were decapitated and the blood was collected from the neck wound. The 2 uterine horns were immediately dissected and fetuses and placenta were excised and weighed.

### Experiment 3

To determine whether the reduction of FFA and TG affects either the oral glucose tolerance test (OGTT) [15] or the intravenous insulin test (IVIT) [15], 2 new groups of pregnant animals were studied, one treated with acipimox (80 mg) and the other with distilled water, as described above for experiment 1. On day 20 of pregnancy, and after a fasting period of 12 hours, an OGTT was performed 3 hours after the administration of acipimox or distilled water, time of maximum decrease in FFA and TG, by administering 2 g glucose/kg body weight dissolved in distilled water by gavage. Blood samples were collected from the tip of the tail into heparinized tubes at 0, 7.5, 15, 22, 30, 60, or 120 minutes after glucose administration. Plasma samples were kept at −20°C until analyzed for glucose and insulin.

In another 2 groups of animals, submitted to the same experimental conditions as for the OGTT, an IVIT was performed 3 hours after the administration of acipimox, through a tail vein, either 10 IU of Actrapid monocomponent porcine insulin (from Novo Industry A/S) dissolved in 1 mL 0.9% NaCl/kg of body weight or 1 mL of 0.9% NaCl/kg. Blood samples were obtained from the tail, before the administration of insulin and 4, 8, 12, 16, and 32 minutes thereafter.

### Blood Collection

Blood was collected in EDTA (1 mg/mL) from the tail or from the open neck after decapitation. The blood was centrifuged at 2500 rpm and plasma stored at −20°C until processed.

### Analysis of the Samples

Glucose, FFA, and TG were determined using commercially available kits: Glucose God-Pad Enzymatic Clorimetric test (Boehringer Mannheim, Mannheim, Germany) for glucose; NEFA C ACS, acid method (Wako Chemical, Neuss, Germany) for FFA; and Triglycerides Enzymatic Trinder method (Menarini Diagnostics, Florence, Italy) for TG. Glycerol was determined using a fluorimetric method [16]. A rat-specific radioimmunoassay kit from Incstar (Stillwater, Minnesota, USA) was used to determine the insulin.

### Statistical Analysis

Means ± SE are given. The significance of the difference between the 2 groups was obtained using the analysis of variance and Tuckey test. When samples from the same animals were studied, a paired *t* test was applied. Statistical analysis was performed using Systat program (Systat, Evanston, Illinois, USA).

### RESULTS

### Experiment 1

In 20-day pregnant animals, 3 hours after the oral administration of distilled water and of the onset of fasting, an increase in plasma FFA and glycerol was observed (Figure 1). However, the administration of acipimox impeded the elevation of both variables at least for 7 hours (Figure 1). Regarding

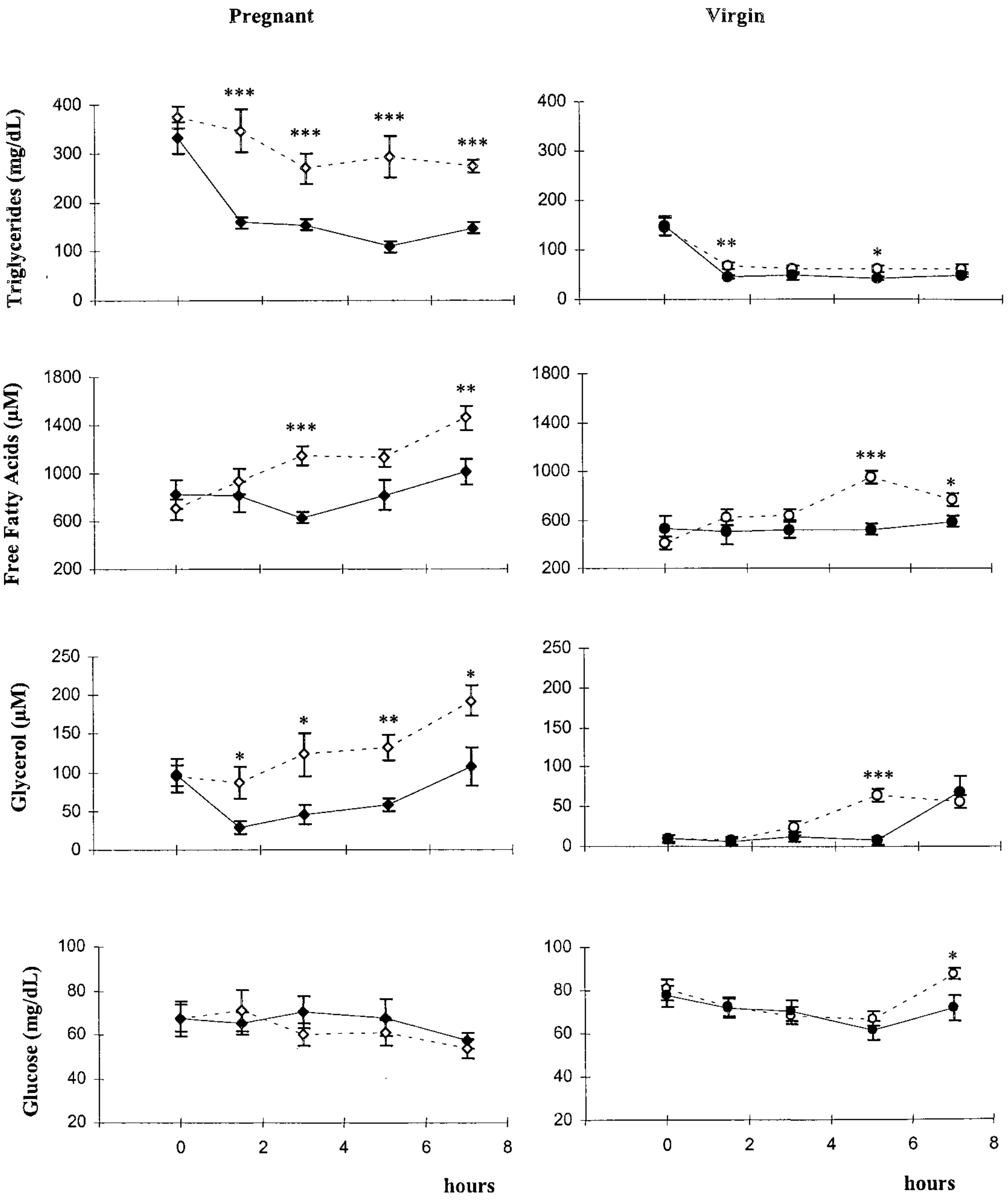

**FIGURE 1**

Effect of acipimox on plasma TG, glycerol, FFA, and glucose in 20-day pregnant and virgin rats. Eighty milligrams of acipimox in 0.3 mL of distilled water were administered by gavage to 20-day pregnant rats and the same amount of distilled water as placebo. Two additional groups of virgin animals were studied under the same experimental conditions. The values are means ± SE of 13 animals. The differences between animals treated with acipimox versus placebo: *$P < .05$; **$P < .01$; ***$P < .001$. Solid lines with solid circles represent animals treated with acipimox; dashed lines with open circles represent the placebo group.

TG, in the group treated with distilled water, plasma concentration of TG remained practically stable during the 7 hours of study (Figure 1). However, in the group treated with acipimox, a marked decrease in plasma concentration of TG was observed after 1.5 hours, and the levels remained lower than in the control group during the 7 hours of the study. In nonpregnant animals, as expected, basal levels (time 0) of FFA, glycerol, and TG were lower than in pregnant animals (Figure 1). In virgin rats receiving distilled water, a rise in plasma levels of FFA and glycerol was observed after 5 hours of the initiation of the study. This effect was not observed in the acipimox-treated group (Figure 1). In either pregnant or virgin animals, the administration of acipimox did not lead to any changes in the plasma concentration of glucose during the 7 hours of the study.

### Experiment 2

Daily administration of acipimox during days 17 to 21 of pregnancy led to a decrease in fetal weight: 4.11 ± 0.12 g in fetuses from rats treated with acipimox versus 4.46 ± 0.21 g in fetus from the control group ($P < .001$). As expected, during the last days of pregnancy in the group receiving distillated water, the concentration of TG and FFA raised steadily with time, whereas no changes were observed in plasma glycerol levels (Table 1). When acipimox was administered every 12 hours during the 17th to the 20th days of pregnancy, an increase in FFA, glycerol, and TG was observed. FFA and glycerol reach levels similar to or even higher than those observed in rats receiving distilled water; the level of TG was slightly lower for acipimox-treated pregnant rats. In virgin animals, the administration of acipimox every 12 hours during 3 days did not have any effect on plasma levels of FFA and glycerol, and decreased the levels of TG compared with values observed in virgin animals treated with distilled water. No changes were found in plasma glucose levels of both pregnant and virgin animals treated with acipimox during the 3 days of the experiment (Table 1). As expected, glucose values of the pregnant rats were lower than in virgin animals (Table 1).

### Experiment 3

We also wanted to determine whether the decrease in plasma concentration of TG and FFA secondary to the administration of acipimox had any effect either in the OGTT or in the IVIT in 20-day pregnant rats, at the time of increased insulin resistance [15]. As can be seen in Figure 2, after the administration of oral glucose, no differences were observed in plasma glucose concentration between control and acipimox groups, in either pregnant or virgin rats. However, in the acipimox-treated animals, a higher increase in plasma insulin was seen after the OGTT in pregnant animals than in the control group, but not in virgin rats (Figure 2). No difference in the plasma glucose concentration after the IVIT was found between the pregnant animals treated with acipimox and the control group (Figure 3).

### DISCUSSION

Both in humans and rats, at the end of pregnancy, there are increased levels of plasma TG and FFA [1–3]. These metabolic substrates can be utilized by most maternal tissues, sparing glucose for glucose-obligatory tissues. In our experiments, as expected, because of the high lipolytic activity [1–3], in 20-day pregnant rats, plasma levels of FFA, glycerol, and TG were higher than in virgin animals. The administration of acipimox to 20-day pregnant rats decreased the plasma levels of FFA, glycerol, and TG by >50%. Thus, our results show that, during

**TABLE 1**

Plasma levels of TG, FFA, glycerol, and glucose in pregnant and virgin rats treated with either acipimox or distilled water

| | Hours after the begining of treatment | Pregnant rats | | Virgin rats | |
|---|---|---|---|---|---|
| | | Acipimox | Placebo | Acipimox | Placebo |
| Triglycerides (mg/dL) | 0 | 250.7 ± 15.5 | 267.1 ± 22.5 | 187.8 ± 17.1 | 175.5 ± 14 |
| | 24 | 253.1 ± 19.8 | 312.1 ± 28.3 | 151.7 ± 19.9 | 170.0 ± 20.6 |
| | 48 | 357.0 ± 35.1* | 391.8 ± 41.2* | 119.3 ± 15** | 145.1 ± 13.4* |
| | 72 | 396.7 ± 41.2** | 459.9 ± 60.6** | 108.5 ± 8.6** | 158.1 ± 7.5 |
| Free fatty acid ($\mu$mol/L) | 0 | 473.2 ± 53.9 | 544.1 ± 45.2 | 477.1 ± 45.6 | 555.9 ± 38.7 |
| | 24 | 934.9 ± 53.5*** | 621.9 ± 25.4 | 833.0 ± 74.6** | 661.5 ± 36.4 |
| | 48 | 1220.9 ± 67.7*** | 832.1 ± 64.8** | 891.5 ± 86.6*** | 668.6 ± 35.7 |
| | 72 | 1239.6 ± 62.4*** | 907.9 ± 91.9*** | 659.3 ± 81.8* | 621.4 ± 26.5 |
| Glycerol ($\mu$mol/L) | 0 | 77.8 ± 9.5 | 97.2 ± 20.1 | 82.6 ± 14.7 | 63.4 ± 14.9 |
| | 24 | 104.4 ± 18.3** | 87.6 ± 19.7 | 93.9 ± 19.9 | 84.6 ± 13.6 |
| | 48 | 122.6 ± 25.5** | 123.3 ± 33.5 | 117.8 ± 21.5* | 70.6 ± 19.9 |
| | 72 | 154.8 ± 26.4*** | 99.0 ± 21.7 | 111.7 ± 20.1* | 127.6 ± 19.8 |
| Glucose (mg/dL) | 0 | 74.4 ± 2.4 | 70.8 ± 3.4 | 90.4 ± 4.4 | 90.9 ± 3.4 |
| | 24 | 71.8 ± 2.8 | 75.6 ± 3.1 | 86.9 ± 4.2 | 87.6 ± 7.1 |
| | 48 | 68.6 ± 2.3 | 69.8 ± 1.6 | 97.2 ± 4.8 | 88.9 ± 3.2 |
| | 72 | 76.1 ± 2.3 | 73.7 ± 2.2 | 112.1 ± 7*** | 103.6 ± 5.5 |

*Note.* Pregnant and virgin rats were treated from days 17 to 20 with either acipimox (80 mg/12 h) or distilled water, as described in "Methods and Experimental Design." Values are means ± SE of 12 animals. Differences between the values observed before the initiation of the treatment and thereafter: $^{*}P < .05$; $^{**}P < .01$; $^{***}P < .001$.

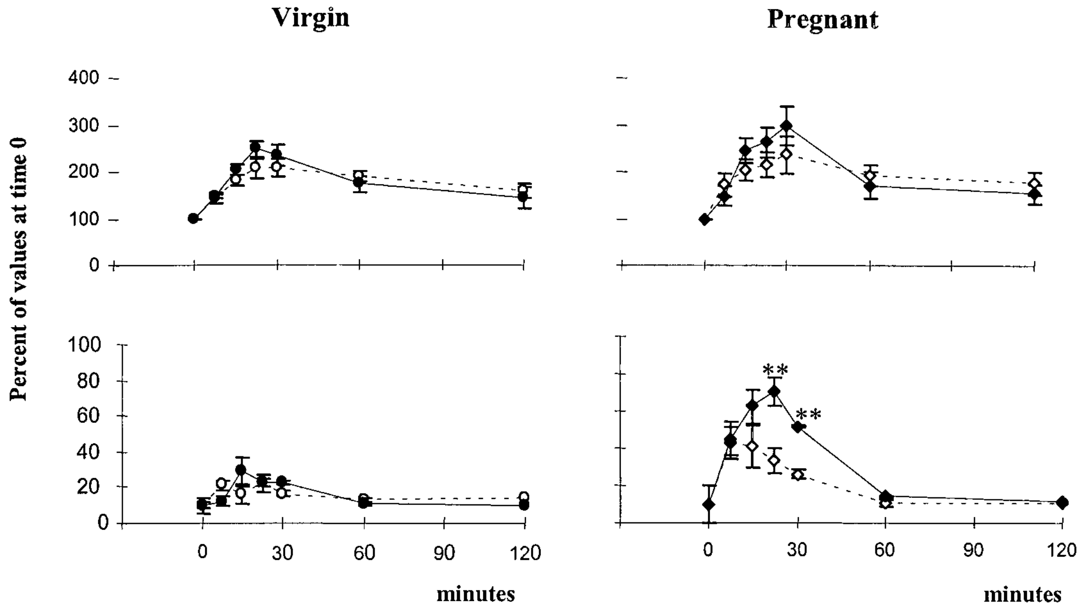

**FIGURE 2**

Results of an OGTT in 20-day pregnant rats treated with a single dose of acipimox or placebo, as described in Figure 1. Two grams of glucose per kilogram body weight were administered 3 hours after receiving the acipimox treatment, when lower levels of FFA and TG are present. Results are presented as percentage of basal levels. Solid lines with solid circles represent the animals treated with acipimox; dashed lines with open circles represent the placebo group. Values are means ± SE of 10 animals. Differences between animals treated with acipimox versus placebo: $^{*}P < .05$; $^{**}P < .01$; $^{***}P < .001$.

pregnancy in rats, acipimox may be used acutely to decrease plasma levels of FFA and TG.

When acipimox was administered every 12 hours during the last 3 days of pregnancy, a rebound effect was observed, as it is

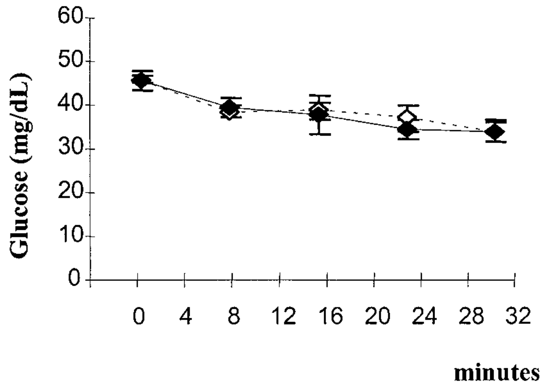

**FIGURE 3**

Under the same conditions described in Figure 2, an IVIT was performed. The same groups as in Figure 2 received either 1 mL/kg body weight of a solution of 10 IU regular insulin/mL or 0.9% NaCl. Solid lines with solid circles represent the animals treated with acipimox and the dashed lines with open circles represent the placebo group. Values are means ± SE of 10 animals. No statistical differences between the 2 groups were found at any time frame.

shown here by the higher levels of FFA and glycerol observed 12 hours after the administration of acipimox, when compared with values observed in the placebo group. This contrasts with no rebound effect observed in virgin animals, when acipimox was given every 12 hours. Similar results have been shown by our group with fenofibrate, another TG-reducing compound [17]. The differences in the rebound effect between pregnant and virgin animals could be secondary to the high lipolytic activity present at the end of gestation, therefore the equilibrium between the inhibitory effects of acipimox on lipolysis are overrun by the lipolytic effects of the high levels of (hPL) or the insulin resistance at late pregnancy [5, 6].

During late gestation, one of the main sources of fuel energy for the maternal tissues are FFA derived from lipolysis of very-low-density lipoprotein (VLDL)-TG [4]. Therefore, the reduction in fetal weight observed on acipimox treatment during the last 3 days of pregnancy could be expected as plasma levels of both FFA and TG decreased. These data are in agreement with the findings in human pregnancies where a positive correlation between the maternal levels of plasma TG and the newborn weight has been observed [10], and the lower weight observed in newborn from pregnancies with a smaller increase in plasma cholesterol through pregnancy [11]. Overall, these results indicate that the elevation of maternal lipid levels through pregnancy is important to allow the maximal fetal growth. In our experiments, we cannot rule out a direct effect of acipimox on fetal growth by crossing the placenta. In fact in human placenta perfusates, acipimox crossed the placenta at a slow rate [18].

Increased levels of FFA and TG have been associated with insulin resistance [19]. In our experiments, 3 hours after the administration of acipimox, despite the reduction by $>50\%$ of both FFA and TG, plasma glucose levels observed after OGTT and IVIT were similar to the values found in the untreated group. Thus, the reduction of TG and FFA, over a short period of time, did not seem to have any effect in the peripheral insulin resistance during late gestation, although we do not know if this is due to changes in insulin sensitivity when FFA and TG are decreased over longer period of time. In type 2 diabetes with increased levels of FFA and TG, the decrease in both by acipimox did not improve peripheral insulin sensitivity [20].

In our experiments, it is remarkable that in late pregnant rats, but not in virgin animals, when insulin resistance is at its highest, the administration of acipimox, which reduced both FFA and TG levels, did increase the glucose-induced insulin release during the OGTT. This result suggests that, during late gestation, the elevated levels of FFA and TG, under certain circumstances, could be toxic to the $\beta$-cell function. In fact, in other conditions prone to the development of type 2 diabetes [21–23], the glucose-induced insulin release is decreased. A similar finding has been observed in pregnant women at risk of developing gestational diabetes [24]. It is attractive to speculate that decreasing FFA and TG, with acipimox, may constitute a therapeutic approach to prevent gestational diabetes.

In conclusion, our results show that the administration of acipimox during the last days of gestation inhibited lipolysis and decreased fetal weight. Over a short period of time, in pregnant rats, the reduction of plasma FFA and TG improved the glucose-induced insulin release but did not seem to have any effect in the peripheral insulin resistance.

### REFERENCES

[1] Knopp, R. H., Bonet, B., Lasunción, A., Montelongo, A., and Herrera, E. (1992) Lipoprotein metabolism in pregnancy. In: *Perinatal Biochemistry*, edited by Herrera, E., and Knopp, R. H., pp. 20–51. Boca Raton, FL, Chemical Rubber Publishing.

[2] Knopp, R. H., Bonet, B., and Zhu, X. (1998) Lipid metabolism in pregnancy. In: *Principles of Perinatal-Neonatal Metabolism*, edited by Cowet, R. M., pp. 221–258. New York, Springer.

[3] Herrera, E., Bonet, B., and Lasunción, M. A. (1998) Maternal-fetal transfer of lipid metabolites. In: *Fetal and Neonatal Physiology*, edited by Polin, R. A., and Fox, W. W., pp. 447–457. Philadelphia, W. B. Saunders.

[4] Freinkel, N. (1980) Banting lecture: Of pregnancy and progeny. *Diabetes,* **29,** 1023–1035.

[5] Catalano, P. M., Tyzibir, E. D., Roman, N. M., Amini, S. B., and Sims, E. A. H. (1991) Longitudinal changes in insulin resistance in non-obese pregnant women. *Am. J. Obstet. Gynecol.,* **165,** 1667–1772.

[6] Desoye, G., Schweditsch, O., Pfeiffer, K. P., Zechner, R., and Kostner, G. M. (1987) Correlation of hormones with lipid and lipoprotein levels during normal pregnancy and postpartum. *J. Clin. Endocrinol. Metab.,* **64,** 704–712.

[7] Sivan, E., Homko, C. J., Whittaker, P. G., Reece, E. A., Chen, X., and Boden, G. (1998) Free fatty acids and insulin resistance during pregnancy. *J. Clin. Endocrin. Metab.,* **83,** 2338–2342.

[8] Howard, B. V. (1987) Lipoprotein metabolism in diabetes mellitus. *J. Lipid Res.,* **28,** 613–628.

[9] Arbogast, B. W., Leeper, S. C., Merrick, R. D., Olive, K. E., and Taylor, R. N. (1994) Which plasma factors bring about disturbance of endothelial function in preeclampsia. *Lancet,* **343,** 340–341.

[10] Knopp, R. H., Magee, M. S., Walden, C. E., Bonet, B., and Benedetti, T. J. (1992) Prediction of infant birthweight by gestational diabetes screening test: Importance of plasma triglyceride. *Diabetes Care,* **15,** 1605–1613.

[11] Sattar, N., Greer, I. A., Galloway, P. J., Packard, C. J., Shepherd, J., Kelly, T., and Mathers, A. (1999) Lipid and lipoprotein concentrations in pregnancies complicated by intrauterine growth restrictions. *J. Clin. Endocrinol. Metab.,* **84,** 128–130.

[12] Christie, A. W., McCormick, D. K. T., Emmison, N., Kraemer, F. B., Alberti, K. G. M. M., and Yeaman, S. J. (1996) Mechanism of anti-lipolytic action of acipimox in isolate rat adipocyte. *Diabetologia,* **39,** 45–53.

[13] Fucella, L. M., Goldaniga, G., Lovisolo, P., Maggi, E., Musatti, L., Mandelli, V., and Sirtoir, C. R. (1980) Inhibition of lipolysis by nicotinic acid and by acipimox. *Clin. Pharmacol. Ther.,* **28,** 790–795.

[14] Shurbaji, A., Berglund, L., and Bjorkhem, I. (1990) The effects of acipimox on triacylglycerol metabolism in rat. *Can. J. Clin. Lab. Invest.,* **50,** 203–208.

[15] Muñoz, C., López-Luna, P., and Herrera, E. (1995) Glucose and insulin tolerance tests in the rat on different days of gestation. *Biol. Neonate,* **68,** 282–291.

[16] Bonet, B., and Herrera, E. (1991) Maternal hypothyroidism during the first half of gestation compromise catabolic adaptations normally occurring during late gestation. *Endocrinology,* **129,** 210–216.

[17] Soria, A., Bocos, C., and Herrera, E. (2002) Opposite metabolic response to fenofibrate treatment in pregnant and virgin rats. *J. Lipid. Res.,* **43,** 74–81.

[18] Ghabrial, H., Czuba, M. A., Stead, C. K., Smallwood, R. A., and Morgan, D. J. (1991) Transfer of acipimox across the isolated perfused human placenta. *Placenta,* **12,** 653–661.

[19] Randle, P. J., Garland, P. B., Hales, C. N., and Newsholme, E. A. (1963) The glucose fatty acid cycle: Its role in insulin sensitivity and the metabolic disturbances of diabetes mellitus. *Lancet,* **1,** 785–789.

[20] Walker, M., Agius, L., Orskov, H., and Alberti, K. G. M. M. (1993) Peripheral and hepatic insulin sensitivity in non-insulin-dependent diabetes mellitus: Effect of nonesterified fatty acids. *Metabolism,* **42,** 601–608.

[21] McGarry, D. J., and Dobbins, R. L. (1999) Fatty acids, lipotoxicity and insulin secretion. *Diabetologia,* **42,** 128–138.

[22] Weyer, C., Bogardus, C., Mott, D. M., and Pratley, R. (1999) The natural history of insulin secretory dysfunction and insulin resistance in the pathogenesis of type 2 diabetes mellitus. *J. Clin. Invest.*, **104,** 787–794.

[23] Paolisso, G., Tagliamonte, M. R., Rizzo, M. R., Gualdiero, P., Saccomanno, F., Gambardella, A., Giugliano, D., D'Onofrio, F., and Howard, B. V. (1998) Lowering fatty acids potentiates acute insulin response in first degree relatives of people with type II diabetes. *Diabetologia,* **41,** 1127–1132.

[24] Xiang, A. H., Peters, R. K., Trigo, E., Kjos, S. L., Lee, W. P., and Buchanan, T. A. (1999) Multiple metabolic defects during late pregnancy in women at high risk for type 2 diabetes. *Diabetes,* **48,** 848–854.

